# Supplementary material for: The effect of peer support on HbA1c levels in middle-aged and elderly patients with type 2 diabetes: a systematic review and meta-analysis
Source: PeerJ. 2025 Aug 28;13:e19803. doi: 10.7717/peerj.19803 (PMC12399075; doi:10.7717/peerj.19803)
Supplement: Supplemental Information 1 [file peerj-13-19803-s001.docx]

**Supplementary Table 1:**

search terms

| **Mesh/Keywords** | **Subject** | |
| --- | --- | --- |
|  | Peer Support | Type 2 Diabetes |
| **Mesh** | -^a)^  - | Type 2 Diabetes Mellitus |
| **Keywords** | Peer Group  Social Support  Peer Influence  Counseling  Peer group  Mental health services  peer relation  peer relations  Peer relationship  peer relationships  peer nomination  peer nominations | Diabetes  Mellitus,Noninsulin-Dependent  Diabetes Mellitus,Ketosis-Resistant  Diabetes Mellitus,Ketosis Resistant  Ketosis-Resistant Diabetes Mellitus  Diabetes Mellitus,Non Insulin Dependent  Diabetes Mellitus,Non-Insulin-Dependent  Non-Insulin-Dependent Diabetes Mellitus  Diabetes Mellitus,Stable  Stable Diabetes Mellitus  Diabetes Mellitus,Type II  NIDDM  Diabetes Mellitus,Noninsulin Dependent  Diabetes Mellitus,Maturity-Onset  Diabetes Mellitus,Maturity Onset  Maturity-Onset Diabetes Mellitus  Maturity Onset Diabetes Mellitus  MODY  Diabetes Mellitus,Slow-Onset  Diabetes Mellitus,Slow Onset  Slow-Onset Diabetes Mellitus  Type 2 Diabetes Mellitus  Noninsulin-Dependent Diabetes Mellitus  Noninsulin Dependent Diabetes Mellitus  Maturity-Onset Diabetes  Diabetes,Maturity-Onset  Maturity Onset Diabetes  Type 2 Diabetes  Diabetes,Type 2  Diabetes Mellitus,Adult-Onset  Adult-Onset Diabetes Mellitus  Diabetes Mellitus,Adult Onset |
| 1. (- for there is no corresponding Mesh) | | |

search strategy(PubMed)

((("Diabetes Mellitus, Type 2"[Mesh]) OR (((((((((((((((((((((((((((Diabetes Mellitus, Noninsulin-Dependent[Title/Abstract]) OR (Diabetes Mellitus, Ketosis-Resistant[Title/Abstract])) OR (Diabetes Mellitus, Ketosis Resistant[Title/Abstract])) OR (Ketosis-Resistant Diabetes Mellitus[Title/Abstract])) OR (Diabetes Mellitus, Non Insulin Dependent[Title/Abstract])) OR (Diabetes Mellitus, Non-Insulin-Dependent[Title/Abstract])) OR (Non-Insulin-Dependent Diabetes Mellitus[Title/Abstract])) OR (Diabetes Mellitus, Stable[Title/Abstract])) OR (Stable Diabetes Mellitus[Title/Abstract])) OR (Diabetes Mellitus, Type II[Title/Abstract])) OR (NIDDM[Title/Abstract])) OR (Diabetes Mellitus, Noninsulin Dependent[Title/Abstract])) OR (Diabetes Mellitus, Maturity-Onset[Title/Abstract])) OR (Diabetes Mellitus, Slow-Onset[Title/Abstract])) OR (Diabetes Mellitus, Slow Onset[Title/Abstract])) OR (Slow-Onset Diabetes Mellitus[Title/Abstract])) OR (Type 2 Diabetes Mellitus[Title/Abstract])) OR (Noninsulin-Dependent Diabetes Mellitus[Title/Abstract])) OR (Noninsulin Dependent Diabetes Mellitus[Title/Abstract])) OR (Maturity-Onset Diabetes[Title/Abstract])) OR (Diabetes, Maturity-Onset[Title/Abstract])) OR (Maturity Onset Diabetes[Title/Abstract])) OR (Type 2 Diabetes[Title/Abstract])) OR (Diabetes, Type 2[Title/Abstract])) OR (Diabetes Mellitus, Adult-Onset[Title/Abstract])) OR (Adult-Onset Diabetes Mellitus[Title/Abstract])) OR (Diabetes Mellitus, Adult Onset[Title/Abstract]))) AND (("Peer Group"[Mesh]) OR ((((((Group, Peer[Title/Abstract]) OR (Groups, Peer[Title/Abstract])) OR (Peer Groups[Title/Abstract])) OR (Counseling[Title/Abstract])) OR (Social Support[Title/Abstract])) OR (Mental Health Services[Title/Abstract])))) AND (randomized controlled trial[Publication Type] OR randomized[Title/Abstract] OR placebo[Title/Abstract])

randomized controlled trial[Publication Type] OR randomized[Title/Abstract] OR placebo[Title/Abstract]

("Peer Group"[Mesh]) OR ((((((Group, Peer[Title/Abstract]) OR (Groups, Peer[Title/Abstract])) OR (Peer Groups[Title/Abstract])) OR (Counseling[Title/Abstract])) OR (Social Support[Title/Abstract])) OR (Mental Health Services[Title/Abstract]))

(((((Group, Peer[Title/Abstract]) OR (Groups, Peer[Title/Abstract])) OR (Peer Groups[Title/Abstract])) OR (Counseling[Title/Abstract])) OR (Social Support[Title/Abstract])) OR (Mental Health Services[Title/Abstract])

"Peer Group"[Mesh]

("Diabetes Mellitus, Type 2"[Mesh]) OR (((((((((((((((((((((((((((Diabetes Mellitus, Noninsulin-Dependent[Title/Abstract]) OR (Diabetes Mellitus, Ketosis-Resistant[Title/Abstract])) OR (Diabetes Mellitus, Ketosis Resistant[Title/Abstract])) OR (Ketosis-Resistant Diabetes Mellitus[Title/Abstract])) OR (Diabetes Mellitus, Non Insulin Dependent[Title/Abstract])) OR (Diabetes Mellitus, Non-Insulin-Dependent[Title/Abstract])) OR (Non-Insulin-Dependent Diabetes Mellitus[Title/Abstract])) OR (Diabetes Mellitus, Stable[Title/Abstract])) OR (Stable Diabetes Mellitus[Title/Abstract])) OR (Diabetes Mellitus, Type II[Title/Abstract])) OR (NIDDM[Title/Abstract])) OR (Diabetes Mellitus, Noninsulin Dependent[Title/Abstract])) OR (Diabetes Mellitus, Maturity-Onset[Title/Abstract])) OR (Diabetes Mellitus, Slow-Onset[Title/Abstract])) OR (Diabetes Mellitus, Slow Onset[Title/Abstract])) OR (Slow-Onset Diabetes Mellitus[Title/Abstract])) OR (Type 2 Diabetes Mellitus[Title/Abstract])) OR (Noninsulin-Dependent Diabetes Mellitus[Title/Abstract])) OR (Noninsulin Dependent Diabetes Mellitus[Title/Abstract])) OR (Maturity-Onset Diabetes[Title/Abstract])) OR (Diabetes, Maturity-Onset[Title/Abstract])) OR (Maturity Onset Diabetes[Title/Abstract])) OR (Type 2 Diabetes[Title/Abstract])) OR (Diabetes, Type 2[Title/Abstract])) OR (Diabetes Mellitus, Adult-Onset[Title/Abstract])) OR (Adult-Onset Diabetes Mellitus[Title/Abstract])) OR (Diabetes Mellitus, Adult Onset[Title/Abstract]))

((((((((((((((((((((((((((Diabetes Mellitus, Noninsulin-Dependent[Title/Abstract]) OR (Diabetes Mellitus, Ketosis-Resistant[Title/Abstract])) OR (Diabetes Mellitus, Ketosis Resistant[Title/Abstract])) OR (Ketosis-Resistant Diabetes Mellitus[Title/Abstract])) OR (Diabetes Mellitus, Non Insulin Dependent[Title/Abstract])) OR (Diabetes Mellitus, Non-Insulin-Dependent[Title/Abstract])) OR (Non-Insulin-Dependent Diabetes Mellitus[Title/Abstract])) OR (Diabetes Mellitus, Stable[Title/Abstract])) OR (Stable Diabetes Mellitus[Title/Abstract])) OR (Diabetes Mellitus, Type II[Title/Abstract])) OR (NIDDM[Title/Abstract])) OR (Diabetes Mellitus, Noninsulin Dependent[Title/Abstract])) OR (Diabetes Mellitus, Maturity-Onset[Title/Abstract])) OR (Diabetes Mellitus, Slow-Onset[Title/Abstract])) OR (Diabetes Mellitus, Slow Onset[Title/Abstract])) OR (Slow-Onset Diabetes Mellitus[Title/Abstract])) OR (Type 2 Diabetes Mellitus[Title/Abstract])) OR (Noninsulin-Dependent Diabetes Mellitus[Title/Abstract])) OR (Noninsulin Dependent Diabetes Mellitus[Title/Abstract])) OR (Maturity-Onset Diabetes[Title/Abstract])) OR (Diabetes, Maturity-Onset[Title/Abstract])) OR (Maturity Onset Diabetes[Title/Abstract])) OR (Type 2 Diabetes[Title/Abstract])) OR (Diabetes, Type 2[Title/Abstract])) OR (Diabetes Mellitus, Adult-Onset[Title/Abstract])) OR (Adult-Onset Diabetes Mellitus[Title/Abstract])) OR (Diabetes Mellitus, Adult Onset[Title/Abstract])

"Diabetes Mellitus, Type 2"[Mesh]

**Supplementary Figure 1:** Forest plots show the effect of peer support on the mean difference in HbA1c (%) by different age patients


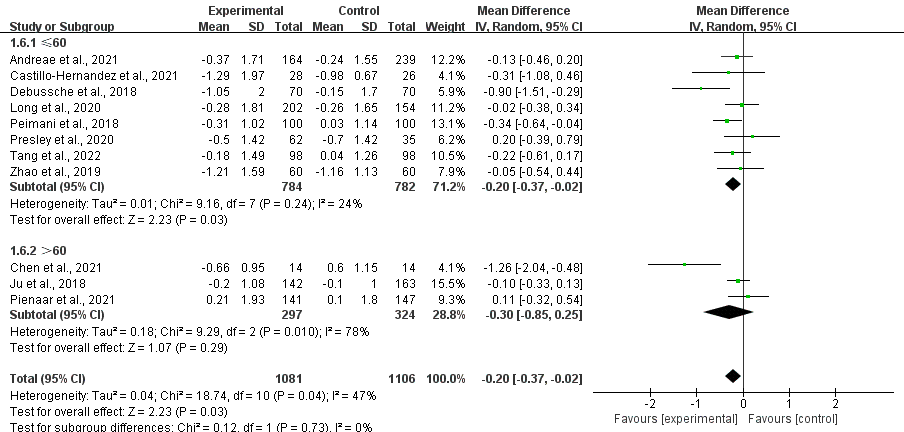


**Supplementary Figure 2:** Forest plots show the effect of peer support on the mean difference in HbA1c (%) by different location


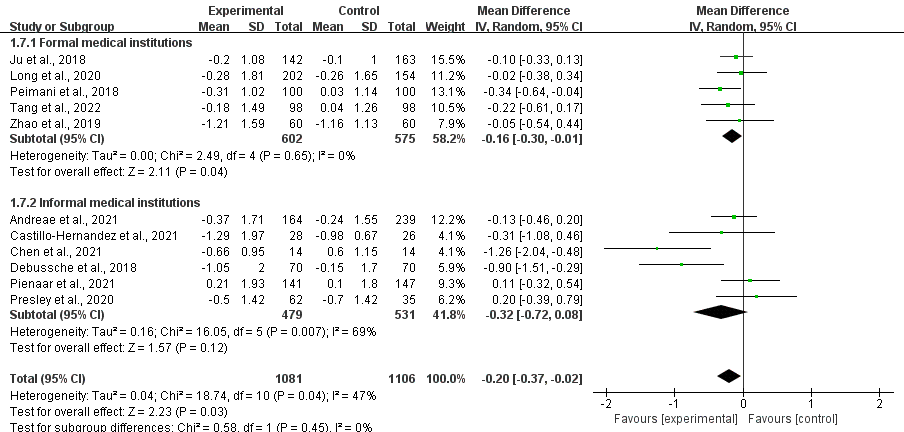


**Supplementary Figure 3:** Forest plots show the effect of peer support on the mean difference in HbA1c (%) by different type of peer support


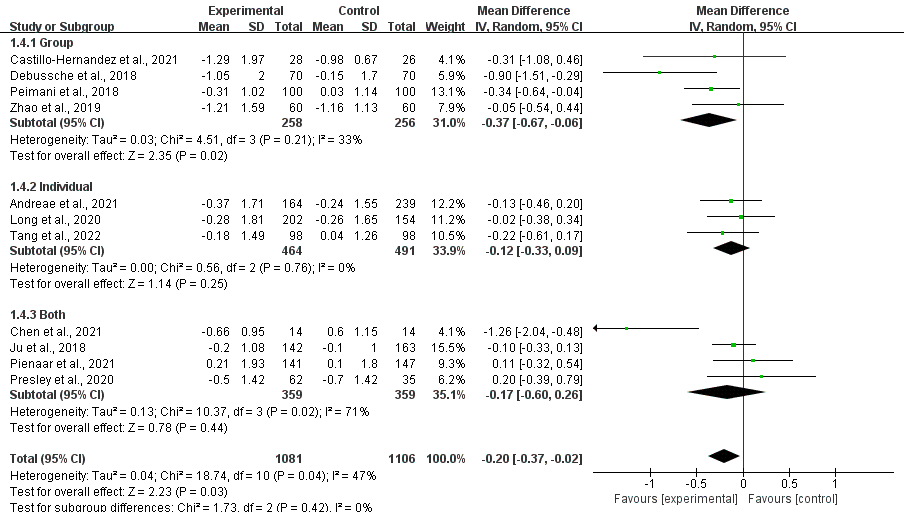


**Supplementary Figure 4:** Forest plots show the effect of peer support on the mean difference in HbA1c (%) by different frequency of peer support


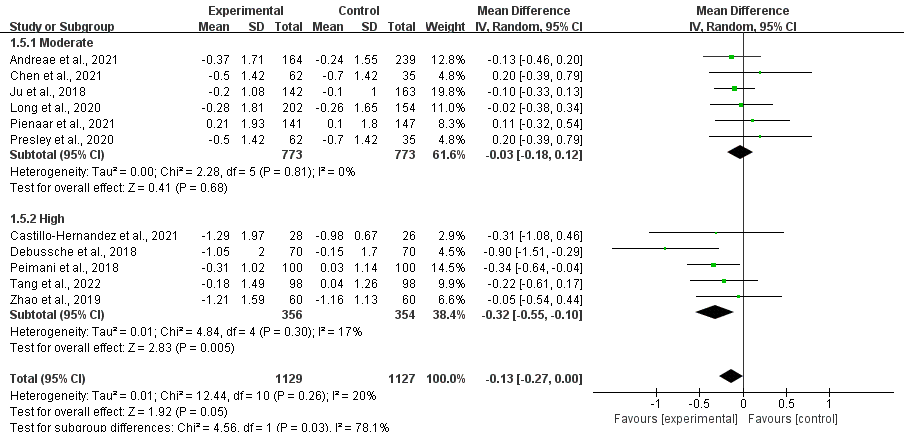


**Supplementary Figure 5:** Forest plots show the effect of peer support on the mean difference in HbA1c (%) by different periods of time


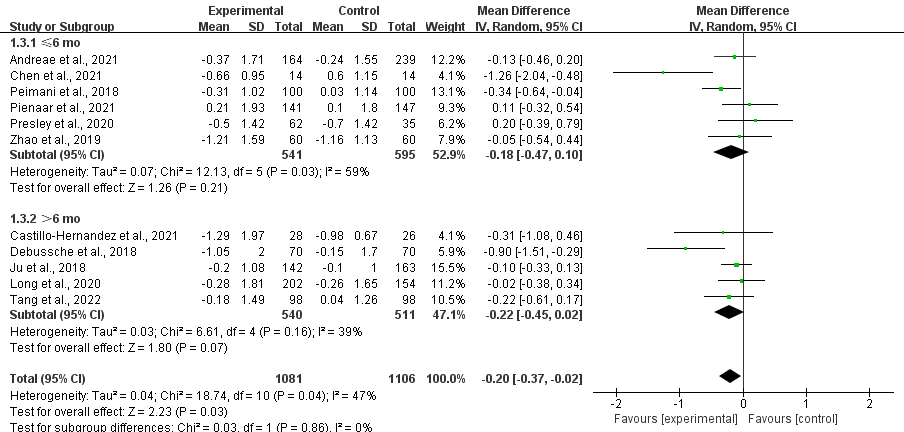


**Supplementary Figure 6:** Funnel plot of Included Studies.

This is a funnel plot with pseudo 95% confidence limits. The plot is a scatter plot with blue dots, where the x-axis is labeled “WMD” and the y-axis is labeled “se(WMD)”. The x-axis ranges from -1.5 to 0.5 and the y-axis ranges from 0 to 0.4. The plot has a dashed line that forms an inverted triangle, and the blue dots are scattered within the triangle, with some outside the triangle.The plot appears to be relatively symmetrical, which suggests that there may not be significant publication bias.


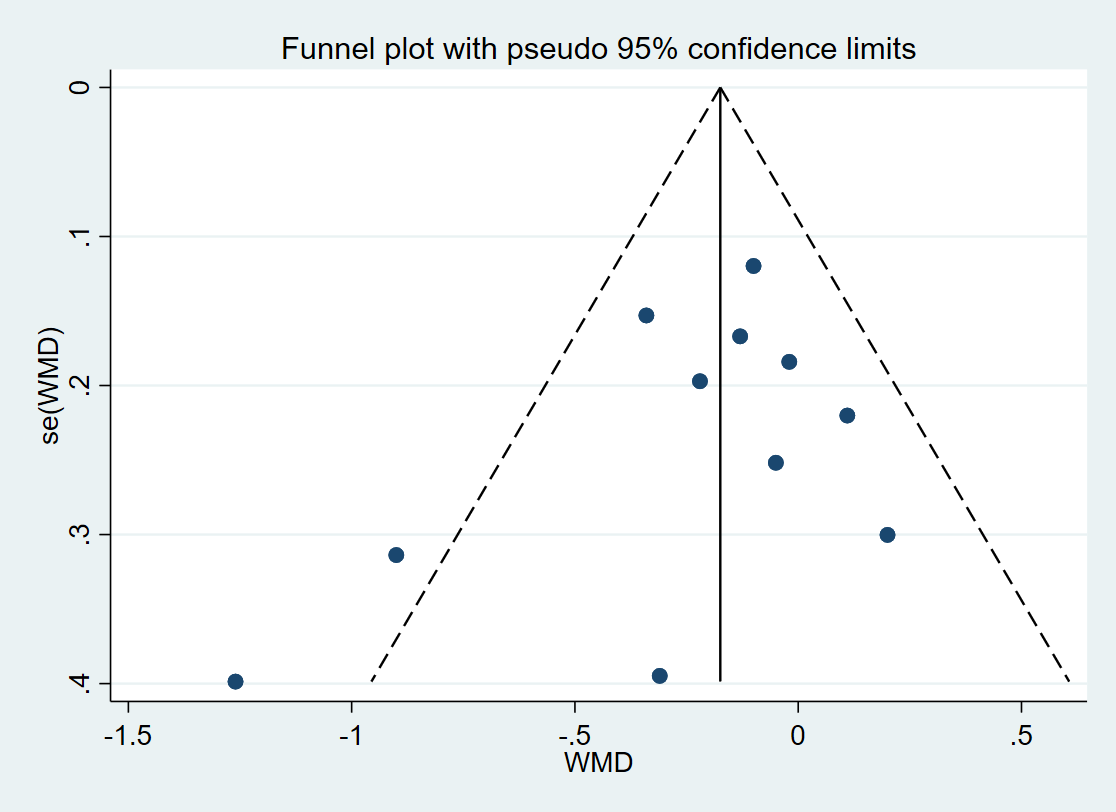


| **Author, Year** | **Study**  **design** | **Patients（I/C）^a)^** | **Mean Age**  **(years,I/C)** | **Peer recruitment and training ^b)^** | **Group/**  **Individual** | **Interventions** | | **control** | **Duration** | **Frequency of contact** | **HbA1c (mean±SD)** |
| --- | --- | --- | --- | --- | --- | --- | --- | --- | --- | --- | --- |
| Ju 2018 | RCT | 163/142 | 67.8/68.6 | R: Diabetes lived with diabetic patients and had good communication skills  Tr: Peer Support Training | Both | Themed and non-themed activities  on diabetes knowledge and skills by  discussion, communication, and  telephone or visit | | 2h diabetes education monthly | 12 mo | Moderate | T: pre/post:6.8±1.5/6.6±1.2,  C: pre/post: 6.7±1.4/6.6±1.0 |
| Castillo 2020 | RCT | 28/26 | 59/56 | R: T2DM patients with A1c <8% and high diabetes Knowledge Questionnaire (DKQ) score  Tr: Peer-themed training weekly for 3 months | Group | Peer-led group meetings weekly and logistics support | | Usual care | 8 mo | High | T: pre/post:8.8±2.2/7.51±1.97  C: pre/post: 8.6±1.8/7.62±0.67 |
|  |  |  |  |  |  | Both I & C:DSMS ^d)^ program:16 1 h weekly group sessions,nutritional counseling and physical activity | | |  |  |  |
| Long 2020 | RCT | 202/154 | 59.6/60.6 | R: T2DM Patients with diabetes and good glycemic control  Tr: 1 h 1-on-1 training session with monthly reinforcement sessions | Individual | Telephone contacts with peers at least weekly and Goal setting | | Usual care | 12 mo | high | T: pre/post:9.3±1.6/9.02±1.81  C: pre/post: 9.8±1.6/9.54±1.65 |
| Peimani 2018 | RCT | 100/100 | 59/58.8 | R: T2DM patients with A1c <8.5% and good interpersonal skills and qualities;Capacity and commitment to undergo the training required etc.  Tr: A 3-day structured, buzz group interactive peers training program developed and conducted by the study team | Both | Peer educators delivered > 2-h education sessions monthly and scheduled telephone contacts weekly | | Usual care | 6 mo | High | T: pre/post:7.29±1.33/6.98±1.31  C: pre/post: 7.47±1.49/9.54±1.44 |
|  |  |  |  |  |  | Both I & C: Four weekly sessions (90 min each) by a credentialed  diabetes educator | | |  |  |  |
| Pienaar 2021 | RCT | 141/147 | 60/62 | R: CHWs ^e)^ had good communication skills  and came from the same communities as the  participants  Tr: 4 months diabetes treatment training course in accordance with Peer Leader Manual | Both | 1h Face-to-face group meetings and home visits monthly | | Health talks,health consultation and medication collection at primary healthcare centre | 4 mo | moderate | T: pre/post:7.86±2.62/8.07±2.4  C: pre/post: 7.77±2.47/7.85±2.09 |
| Andreae 2021 | RCT | 164/239 | 58.12/56.5 | R: Diabetes came from the same communities as the participants  Tr:NR ^c)^ | Individual | Diabetes education video lessons and one-on-one telephonic peer coaching | | A self-paced general health education program and checked by telephone calls | 6 mo | Moderate | T: pre/post:8.4±2.11/8.1±1.9  C: pre/post: 8.3±1.99/8.1±1.8 |
| Tang 2022 | RCT | 98/98 | 60.5/58.5 | R: T2DM patients with A1c <8.5%; adherent  to treatment and behavior change regimens;  Tr: 30-h peer supporter training program to equip trainees with the diabetes knowledge, facilitation strategies, behavior modification techniques, and communication skills | Individual | 12 weekly contacts (face-to-face and telephone support)in the first 3 months and 18 biweekly telephone support contacts over the last 9 months | | Usual care | 12 mo | High | T: pre/post:8.03±1.82/7.85±1.49  C: pre/post: 8.16±1.61/8.2±1.26 |
| Chen 2021 | RCT | 14/14 | 71.71/72.79 | R: Diabetic Volunteers with good leadership quality and self-management ability  Tr: 3h weekly peer support training sessions and the self management skills training examination | Group | Peer-led diabetes self-management programs:1.5h education sessions and group activity per week for 4 weeks and telephone support three times per week | | Self-management manual and  usual Care | 5mo | High | T: pre/post:8.17±1.26/7.51±1.2  C: pre/post: 8.21±1.25/8.81±1.6 |
| Zhao 2019 | RCT | 60/60 | 52.88/52.05 | R: T2DM who had an HbA1c level of less than 8.5%; adherent to treatment and behavior change regimens;  Tr: 1 h training program weekly for 2 months | Group | Formation of peer education groups on ‘We Chat’, emotional support and daily reminders of visits and reviews | | Training courses which included basic medical knowledge and management skills of diabetes | 6 mo | High | T: pre/post:7.97±2.13/6.76±1.01  C: pre/post: 7.81±1.77/6.65±0.79 |
| Presley 2020 | RCT | 62/35 | 54.6/55.5 | R: T2DM or having lived with someone  diagnosed with diabetes and had completed  some college or a college degree.  Tr: Peer support training sessions | Both | Peer support provided by CHWs using a mHealth web; weekly calls for 3 months then monthly calls for 3 months; face-to-face visits with peer supporters were scheduled every month | usual care | | 6 mo | High | T: pre/post:10.1±1.7/9.6±1.9  C: pre/post: 9.8±1.7/9.1±1.9 |
|  |  |  |  |  |  | Both I & C: community-based DSME course | | |  |  |  |
| Debussche 2018 | RCT | 70/70 | 53.9/51.1 | R: Peer leaders with diabetes came from the same communities as the participants had good communication skills and delivered educational sessions  Tr: 4-day training program on how to facilitate  and structure the different cardiovascular risk management sessions | Group | Peer-delivered 12 group sessions of 90–120 min on different topics for 12 months | | usual care | 12 mo | Moderate | T: pre/post: 10.6±1.8/9.55±2.1  C:pre/post:10.8±1.97/10.65±1.55 |

**Supplementary Table 2:** **Characteristics of studies included in this systematic review**

**Supplementary Table 3:** PRISMA Checklist for systematic review and meta-analysis

| **Section and Topic** | **Item #** | **Checklist item** | **Location where item is reported** |
| --- | --- | --- | --- |
| **TITLE** | | |  |
| Title | 1 | Identify the report as a systematic review. | PAGE 1 |
| **ABSTRACT** | | |  |
| Abstract | 2 | See the PRISMA 2020 for Abstracts checklist. | PAGE 1 |
| **INTRODUCTION** | | |  |
| Rationale | 3 | Describe the rationale for the review in the context of existing knowledge. | PAGE 1 |
| Objectives | 4 | Provide an explicit statement of the objective(s) or question(s) the review addresses. | PAGE 1-2 |
| **METHODS** | | |  |
| Eligibility criteria | 5 | Specify the inclusion and exclusion criteria for the review and how studies were grouped for the syntheses. | PAGE 3 |
| Information sources | 6 | Specify all databases, registers, websites, organisations, reference lists and other sources searched or consulted to identify studies. Specify the date when each source was last searched or consulted. | PAGE 2-3 |
| Search strategy | 7 | Present the full search strategies for all databases, registers and websites, including any filters and limits used. | PAGE 2-3 |
| Selection process | 8 | Specify the methods used to decide whether a study met the inclusion criteria of the review, including how many reviewers screened each record and each report retrieved, whether they worked independently, and if applicable, details of automation tools used in the process. | PAGE 3 |
| Data collection process | 9 | Specify the methods used to collect data from reports, including how many reviewers collected data from each report, whether they worked independently, any processes for obtaining or confirming data from study investigators, and if applicable, details of automation tools used in the process. | PAGE 3 |
| Data items | 10a | List and define all outcomes for which data were sought. Specify whether all results that were compatible with each outcome domain in each study were sought (e.g. for all measures, time points, analyses), and if not, the methods used to decide which results to collect. | PAGE 3-4 |
|  | 10b | List and define all other variables for which data were sought (e.g. participant and intervention characteristics, funding sources). Describe any assumptions made about any missing or unclear information. | PAGE 3-4 |
| Study risk of bias assessment | 11 | Specify the methods used to assess risk of bias in the included studies, including details of the tool(s) used, how many reviewers assessed each study and whether they worked independently, and if applicable, details of automation tools used in the process. | PAGE 3-4 |
| Effect measures | 12 | Specify for each outcome the effect measure(s) (e.g. risk ratio, mean difference) used in the synthesis or presentation of results. | PAGE 4 |
| Synthesis methods | 13a | Describe the processes used to decide which studies were eligible for each synthesis (e.g. tabulating the study intervention characteristics and comparing against the planned groups for each synthesis (item #5)). | N/A |
|  | 13b | Describe any methods required to prepare the data for presentation or synthesis, such as handling of missing summary statistics, or data conversions. | N/A |
|  | 13c | Describe any methods used to tabulate or visually display results of individual studies and syntheses. | PAGE 4 |
|  | 13d | Describe any methods used to synthesize results and provide a rationale for the choice(s). If meta-analysis was performed, describe the model(s), method(s) to identify the presence and extent of statistical heterogeneity, and software package(s) used. | PAGE 4-5 |
|  | 13e | Describe any methods used to explore possible causes of heterogeneity among study results (e.g. subgroup analysis, meta-regression). | PAGE 5 |
|  | 13f | Describe any sensitivity analyses conducted to assess robustness of the synthesized results. | PAGE 6 |
| Reporting bias assessment | 14 | Describe any methods used to assess risk of bias due to missing results in a synthesis (arising from reporting biases). | PAGE 4-5 |
| Certainty assessment | 15 | Describe any methods used to assess certainty (or confidence) in the body of evidence for an outcome. | PAGE 5 |
| **RESULTS** | | |  |
| Study selection | 16a | Describe the results of the search and selection process, from the number of records identified in the search to the number of studies included in the review, ideally using a flow diagram. | PAGE 4 |
|  | 16b | Cite studies that might appear to meet the inclusion criteria, but which were excluded, and explain why they were excluded. | N/A |
| Study characteristics | 17 | Cite each included study and present its characteristics. | PAGE 4-6 |
| Risk of bias in studies | 18 | Present assessments of risk of bias for each included study. | PAGE 4-5 |
| Results of individual studies | 19 | For all outcomes, present, for each study: (a) summary statistics for each group (where appropriate) and (b) an effect estimate and its precision (e.g. confidence/credible interval), ideally using structured tables or plots. | PAGE 5 |
| Results of syntheses | 20a | For each synthesis, briefly summarise the characteristics and risk of bias among contributing studies. | PAGE 4-5 |
|  | 20b | Present results of all statistical syntheses conducted. If meta-analysis was done, present for each the summary estimate and its precision (e.g. confidence/credible interval) and measures of statistical heterogeneity. If comparing groups, describe the direction of the effect. | PAGE 5 |
|  | 20c | Present results of all investigations of possible causes of heterogeneity among study results. | PAGE 5 |
|  | 20d | Present results of all sensitivity analyses conducted to assess the robustness of the synthesized results. | PAGE 6 |
| Reporting biases | 21 | Present assessments of risk of bias due to missing results (arising from reporting biases) for each synthesis assessed. | PAGE 4-5 |
| Certainty of evidence | 22 | Present assessments of certainty (or confidence) in the body of evidence for each outcome assessed. | PAGE 5 |
| **DISCUSSION** | | |  |
| Discussion | 23a | Provide a general interpretation of the results in the context of other evidence. | PAGE 6 |
|  | 23b | Discuss any limitations of the evidence included in the review. | PAGE 6 |
|  | 23c | Discuss any limitations of the review processes used. | PAGE 6-7 |
|  | 23d | Discuss implications of the results for practice, policy, and future research. | PAGE 6-7 |
| **OTHER INFORMATION** | | |  |
| Registration and protocol | 24a | Provide registration information for the review, including register name and registration number, or state that the review was not registered. | Registered on International Prospective Register of Systematic Reviews (PROSPERO) and the registration number is CRD42023462231 |
|  | 24b | Indicate where the review protocol can be accessed, or state that a protocol was not prepared. | N/A |
|  | 24c | Describe and explain any amendments to information provided at registration or in the protocol. | N/A |
| Support | 25 | Describe sources of financial or non-financial support for the review, and the role of the funders or sponsors in the review. |  |
| Competing interests | 26 | Declare any competing interests of review authors. | PAGE 3-4 |
| Availability of data, code and other materials | 27 | Report which of the following are publicly available and where they can be found: template data collection forms; data extracted from included studies; data used for all analyses; analytic code; any other materials used in the review. | PAGE 3-4 |
