# Supplementary material for: The effect of peer support on HbA1c levels in middle-aged and elderly patients with type 2 diabetes: a systematic review and meta-analysis
Source: PeerJ. 2025 Aug 28;13:e19803. doi: 10.7717/peerj.19803 (PMC12399075; doi:10.7717/peerj.19803)
Supplement: Supplemental Information 2 [file peerj-13-19803-s002.docx]

**Target Audience**

This systematic review and meta-analysis is primarily intended for a diverse audience that includes practicing clinicians, diabetes care specialists, public health policymakers, and researchers in the field of diabetes management. Our study aims to provide these stakeholders with comprehensive insights into the efficacy of peer support interventions on glycemic control, specifically focusing on HbA1c levels among middle-aged and elderly patients with Type 2 diabetes. By presenting a synthesis of current evidence, we hope to inform clinical practice, guide the development of diabetes management strategies, and influence policy decisions that enhance the incorporation of peer support programs in healthcare settings. Additionally, this review serves as a valuable resource for academic researchers seeking to explore further the mechanisms underlying the benefits of peer support and to identify gaps for future investigation.
